# Correlation between the expression levels of miR-199a-3p and FN1 in serum and aqueous humor and the severity of type 2 diabetic retinopathy in a Chinese population

**DOI:** 10.1186/s12886-026-04838-1

**Published:** 2026-04-28

**Authors:** Yang Chen, Nianlin Chen, Xiangrui Meng, Minli Linghu, Yikeng Huang, Xionggao Huang

**Affiliations:** 1 Department of Ophthalmology, The First Affiliated Hospital of Hainan Medical University, Haikou, Hainan, 570102 China; 2 Key Laboratory of Emergency and Trauma of Ministry of Education, Department of Emergency Surgery, Key Laboratory of Hainan Trauma and Disaster Rescue, The First Affiliated Hospital, Hainan Medical University, Haikou, 570102 China; 3 Departments of Ophthalmology, Shanghai General Hospital, Shanghai, 200000 China

**Keywords:** Type 2 diabetic retinopathy, microRNA-199a-3p, Fibronectin 1, Serum, Aqueous humor, Biomarker

## Abstract

**Background:**

To investigate the correlation between the expression levels of microRNA-199a-3p (miR-199a-3p) and Fibronectin 1 (FN1) in serum and aqueous humor and the severity of Type 2 Diabetic Retinopathy (DR) in a Chinese population.

**Methods:**

The dataset GSE102485 (containing 3 normal controls and 22 DR samples) was downloaded from the Gene Expression Omnibus (GEO) database as a discovery set, and the dataset GSE60436 (containing 3 normal controls and 6 DR samples) was used as a validation set. The limma package was employed for differential expression analysis to identify differentially expressed genes (DEGs). The potential downstream target genes of miR-199a-3p were predicted using seven databases including ENCORI. A Venn diagram was used to identify the intersection between the DEGs and the predicted miR-199a-3p target genes. Gene Ontology (GO) and Kyoto Encyclopedia of Genes and Genomes (KEGG) pathway enrichment analyses were performed on the intersecting genes. A protein-protein interaction (PPI) network was constructed, and the CIBERSORT algorithm was applied for in-depth analysis of FN1 expression characteristics. Furthermore, the expression pattern of FN1 was validated in independent datasets concerning its association with miR-199a-3p and DR. Finally, real-time quantitative polymerase chain reaction (qPCR) was used to verify the relationship between the expression levels of miR-199a-3p and FN1 in clinical serum and aqueous humor samples and DR severity.

**Results:**

Bioinformatics analysis identified a total of 458 DEGs, comprising 214 upregulated and 244 downregulated genes. A heatmap was generated for the top 50 most significantly altered genes. Venn diagram analysis revealed three overlapping genes (PHYHIPL, FN1, CALD1) between the DEGs from GSE102485 and the predicted miR-199a-3p target genes. Further analysis using the miRDB website indicated that FN1 had the strongest correlation with miR-199a-3p, suggesting both as potential important biomarkers for DR. Additionally, the relative expression level of serum miR-199a-3p was significantly negatively correlated with fasting plasma glucose (FPG) (*r* = -0.425, *P* = 0.012), glycated hemoglobin (HbA1c) (*r* = -0.513, *P* = 0.003), and duration of diabetes (*r* = -0.587, *P* < 0.001), indicating its decrease with elevated blood glucose and prolonged disease course. Conversely, serum FN1 levels showed significant positive correlations with FPG (*r* = 0.458, *P* = 0.008), HbA1c (*r* = 0.621, *P* < 0.001), and diabetes duration (*r* = 0.694, *P* < 0.001), suggesting that poor glycemic control and disease progression may promote FN1 overexpression. Both biomarkers demonstrated good diagnostic value for DR, with miR-199a-3p exhibiting the highest diagnostic efficacy.

**Conclusion:**

The expression levels of miR-199a-3p and FN1 in serum and aqueous humor are significantly correlated with the severity of Type 2 Diabetic Retinopathy, highlighting their potential as non-invasive diagnostic and prognostic biomarkers for DR.

## Background

Diabetic retinopathy (DR) is one of the most common and severe microvascular complications of diabetes mellitus (DM), and it has become the leading cause of visual impairment and blindness in the working-age population worldwide [1]. Its pathological process involves neurovascular unit damage induced by chronic hyperglycemia, including vascular endothelial dysfunction, blood-retinal barrier disruption, neurodegeneration, and ultimately pathological neovascularization [2]. Despite the availability of various detection and intervention strategies to date, the achievement of early accurate diagnosis and effective prevention of DR remains an urgent unmet clinical need. Accordingly, the identification of sensitive and specific biomarkers is of great significance for facilitating the early recognition, disease monitoring and targeted therapy of DR.

From an epidemiological perspective, the prevalence of DR is closely associated with the duration of DM, as well as multiple metabolic parameters such as glycemic control status, blood pressure and dyslipidemia [3, 4]. Recent studies have demonstrated that more than 40% of patients with DM suffer from DR of varying severity, among which diabetic macular edema and proliferative DR are the most critical subtypes, both of which significantly increase the risk of visual loss [5]. In addition, a number of large-scale cohort studies in recent years have revealed the correlation between serum biomarkers and the development and progression of DR, highlighting the regulatory role of microRNAs (miRNAs) and their target genes in diabetic microangiopathy. These findings indicate an urgent practical need for in-depth molecular investigations to elucidate the pathogenic mechanisms underlying DR [6].

Currently, research on DR biomarkers is accumulating, but most studies focus on single molecules or signaling pathways, and systematic investigations into the interaction and regulatory network of miRNAs and their target genes in DR are still lacking. In particular, miR-199a-3p, a pleiotropic miRNA, exerts biological effects such as regulating cell proliferation, inflammation and fibrosis in various diseases, yet its expression characteristics and functional roles in DR have not been fully elucidated [7]. Our preliminary studies have shown that miR-199a-3p modulates the PI3K/AKT signaling pathway by targeting vascular endothelial growth factor (VEGF) in in vitro DR models, thereby inhibiting high glucose-induced proliferation, migration and angiogenesis of human retinal microvascular endothelial cells (hRMECs) and promoting their apoptosis. The expression of miR-199a-3p is significantly downregulated under high glucose conditions, while VEGF expression is upregulated; furthermore, the PI3K inhibitor LY294002 can enhance the anti-angiogenic effect of miR-199a-3p. These results suggest that miR-199a-3p may serve as a potential therapeutic target associated with angiogenesis in DR [8]. On the other hand, fibronectin 1 (FN1), a key component of the extracellular matrix, is involved in tissue remodeling and fibrogenesis, and is closely associated with fibrosis and inflammatory responses in various pathological conditions [9, 10]. Emerging evidence has indicated the potential role of FN1 in diabetes-related complications, but its specific expression pattern and regulatory mechanism in DR remain to be further clarified. Therefore, investigating the expression changes and interaction between miR-199a-3p and FN1 is expected to unravel the molecular pathogenic mechanisms of DR and provide novel diagnostic and therapeutic targets for clinical practice.

This study innovatively focuses on the expression characteristics and correlation of miR-199a-3p and its potential target gene FN1 in DR, and systematically analyzes their potential roles in DR progression by combining bioinformatics analysis with clinical sample validation. By integrating data from public gene expression databases, along with miRNA target gene prediction and functional enrichment analysis, the present study aims to comprehensively elucidate the regulatory network and biological significance of miR-199a-3p and FN1 in DR, thereby providing a new perspective for the screening of molecular biomarkers and mechanistic research of DR. In addition, this study is designed to fill the research gap in the investigation of miRNAs and their target genes in the field of DR, and to promote the clinical translation of early diagnosis and precise intervention for DR.

In terms of research methods, multiple datasets from the Gene Expression Omnibus (GEO) database were employed in this study, and the limma R package was used to perform differential expression analysis for the identification of DR-related differentially expressed genes (DEGs) and differentially expressed miRNAs (DEMs). Meanwhile, authoritative databases such as ENCORI were utilized to predict the target genes of miR-199a-3p, and Venn diagram analysis was conducted to screen FN1 as the key target gene. Subsequently, Gene Ontology (GO) and Kyoto Encyclopedia of Genes and Genomes (KEGG) pathway enrichment analyses were performed to systematically elucidate the biological functions and involved signaling pathways of the differentially expressed genes. Finally, combined with clinical samples, quantitative real-time polymerase chain reaction (qRT-PCR) was used to detect the expression levels of miR-199a-3p and FN1 in serum and aqueous humor, so as to further evaluate their associations with the severity of DR. This methodological system integrates data mining and experimental validation, which not only ensures the systematicity of the study but also enhances the clinical relevance of the results.

In summary, the present study aims to investigate the expression characteristics and interaction of miR-199a-3p and its target gene FN1 in DR through systematic bioinformatics analysis and clinical validation. It is expected to provide novel molecular clues for unraveling the pathogenic mechanisms of DR, and to lay a theoretical foundation for the development of strategies for early diagnosis and targeted therapy of DR.

## Methods

### Processing of GEO datasets

The training dataset GSE102485 [11] and validation datasets GSE60436 [12] were downloaded from the Gene Expression Omnibus (GEO) database (https://www.ncbi.nlm.nih.gov/). GSE102485 was based on the Affymetrix^®^ GPL18573 platform, including 3 normal samples and 22 DR samples; GSE60436 was generated on the Affymetrix^®^ GPL6884 platform, consisting of 3 normal samples and 6 DR samples.

### Screening of differentially expressed genes

The R package limma (v3.56.2) was applied to perform differential expression analysis for identifying differentially expressed genes (DEGs). Volcano plots were plotted using the ggplot2 package (v3.4.3), and heatmaps were generated with the pheatmap package (v1.0.12). Genes were ranked by log fold change (log_2_FC), and the screening criteria for DEGs were set as |log_2_FC| > 1.5 and *P* < 0.05, with false discovery rate (FDR) correction performed to control for Type I errors caused by multiple testing. The top 25 significantly upregulated and 25 significantly downregulated genes (after FDR correction) were selected for heatmap construction.

### Target gene screening and intersection analysis

The downstream target genes of miR-199a-3p were predicted using seven authoritative databases, including ENCORI (https://rnasysu.com/encori/index.php), miRDB (https://mirdb.org/), miRWalk (http://mirwalk.umm.uni-heidelberg.de/), RNA22 (https://cm.jefferson.edu/rna22/), RNAInter (http://www.rna-society.org/rnainter/), TargetMiner (https://www.hsls.pitt.edu/obrc/index.php?page=URL1255462522) and TargetScan (https://www.targetscan.org/). The R package VennDiagram (v1.7.3) was utilized to obtain the overlapping genes between DEGs and the predicted target genes of miR-199a-3p.

### Box plots and receiver operating characteristic (ROC) curves

To further investigate the gene expression differences between the DR and normal groups, differential box plots of hub genes were generated using the ggboxplot function in both the training and validation datasets, with distinct colors used to distinguish the DR and normal groups. In addition, the stat_compare_means function was added to perform statistical analysis of intergroup differences, and significance levels were marked with symbolic annotations. For the evaluation of diagnostic efficiency, the area under the curve (AUC) of the receiver operating characteristic (ROC) curve was calculated, and ROC curves were plotted using the R package pROC (v1.18.4).

### Functional enrichment

DR patients were divided into FN1 high-expression and low-expression groups based on the median expression level of FN1. Differential expression analysis was performed between the two groups, and Gene Set Enrichment Analysis (GSEA), Gene Ontology (GO), and Kyoto Encyclopedia of Genes and Genomes (KEGG) analyses were conducted to explore the underlying molecular mechanisms.

GO analysis was performed to annotate biological functions across three categories: biological process (BP), cellular component (CC), and molecular function (MF). KEGG pathway analysis was used to identify the key signaling pathways involved. All enrichment analyses were carried out using the R package clusterProfiler (v4.8.3).

In addition, differential box plots of hub genes in the training and validation datasets were generated using the ggboxplot function, with distinct colors distinguishing DR and normal groups. The stat_compare_means function was applied for intergroup statistical comparisons, and significance levels were labeled with symbolic annotations.

### Immune infiltration

The CIBERSORT algorithm was used to explore the immune characteristics of the DR and normal groups. Violin plots were generated with the ggplot2 to visualize the infiltration profiles of 22 immune cell types and observe the differences in immune cell infiltration between the DR and normal groups. Subsequently, Spearman’s rank correlation analysis was performed to further investigate the correlation between FN1 expression and immune cell infiltration.

### Construction and analysis of protein-protein interaction (ppi) network

To systematically explore the potential interacting partners of fibronectin 1 (FN1) and the biological networks it participates in, a protein-protein interaction (PPI) network was constructed using the STRING database (Search Tool for the Retrieval of Interacting Genes/Proteins, v12.0; https://cn.string-db.org/).

### Study subjects

A total of 10 patients with non-proliferative diabetic retinopathy (NPDR) and 10 patients with proliferative diabetic retinopathy (PDR) who were diagnosed at the First Affiliated Hospital of Hainan Medical University from January 2025 to September 2025 were enrolled as the study groups. Meanwhile, 20 patients with type 2 diabetes mellitus (T2DM) without DR and 20 cataract patients without DM during the same period were recruited as the control groups. Among all subjects, 19 were male and 21 were female, with ages ranging from 50 to 80 years. Basic clinical data of all subjects were collected, including body mass index (BMI), fasting blood glucose (FBG), glycosylated hemoglobin (HbA1c), triglycerides (TG) and duration of diabetes.

Inclusion criteria: ①Conforming to the diagnostic criteria for T2DM formulated by the American Diabetes Association (ADA); ②Achieving normoglycemic control.

Exclusion criteria: ① Suffering from acute or chronic inflammatory diseases; ② Having youth-onset diabetes, type 1 diabetes mellitus or mitochondrial diabetes; ③ Complicated with systemic diseases.

Fasting elbow venous blood (6 mL) was collected from all subjects and centrifuged at 3000 r/min for 10 min; the upper serum layer was aspirated, stored in EP tubes and preserved at -80 ℃ for subsequent detection. Approximately 0.15 mL of aqueous humor was collected from each patient via clear corneal paracentesis using a 30-gauge needle, with the needle carefully avoiding the iris, cornea and anterior lens capsule. Aqueous humor samples were collected in sterile EP tubes and processed within 2 h; the samples were centrifuged at 3000 r/min for 10 min to prevent contamination by cells/cellular debris, and the supernatant was aspirated and stored at -80 ℃.This research plan has been approved by the Medical Ethics Committee of the First Affiliated Hospital of Hainan Medical University (Approval Number: 2025-KYL-165). Before including the subjects in the study, all participants have given written consent. This trial strictly follows the guidelines for clinical trials on human subjects and the “Helsinki Declaration”.

### Quantitative real-time polymerase chain reaction (qRT-PCR)

Stem-loop reverse transcription was adopted for the reverse transcription of miR-199a-3p, and total RNA was extracted from samples using Trizol reagent (Invitrogen, USA) following the manufacturer’s instructions. The quality of extracted RNA was verified by 1% agarose gel electrophoresis (to confirm the integrity of 18 S and 28 S rRNA bands) and a Nanodrop 2000 spectrophotometer (Thermo Fisher Scientific, USA), with an A260/A280 ratio between 1.8 and 2.0 considered to indicate high-quality RNA. Total RNA was reverse-transcribed into complementary DNA (cDNA) as the amplification template. For internal reference selection, U6 snRNA was chosen as the internal reference for miR-199a-3p (due to its stable expression in various cell types and resistance to experimental conditions), and GAPDH was selected as the internal reference for FN1 (a widely used housekeeping gene with stable expression in tissues and cells involved in this study). All qPCR experiments were performed in three technical replicates and three biological replicates to ensure reproducibility.

The specific reverse transcription primer sequences were as follows: miR-199a-3p forward primer: 5´-GGTGCAGGGTC CGAGGTAT-3´, reverse primer: 5´-GGCGGACAGTAGTCTGCACAT-3´; U6 (internal reference) forward primer: 5´-GCTTCGGCAGCACATATACTAAAAT-3´, reverse primer: 5´-CGCTTCACGAATTTGCGTGTCAT-3´. For FN1, total RNA was reverse-transcribed into cDNA as the amplification template, with the specific primer sequences: FN1 forward primer: 5´-TGGGCAACTCTGTCAACGAA-3´, reverse primer: 5´-GAGCAAATGGCACCGAGATA-3´; GAPDH (internal reference) forward primer: 5´-GGATTTGGTCGTATTGGG-3´, reverse primer: 5´-GGAAGATGGTGATGGGATT-3´. All primers were designed and synthesized by Wuhan Servicebio Technology Co., Ltd.

qRT-PCR reactions were performed using cDNA and SYBR Green dye (Applied Biosystems, USA) in a total reaction volume of 20.0 µL, which contained 4.0 µL of ddH2O, 1.5 µL each of the forward and reverse primers (10.0 µmol/L), 2.0 µL of cDNA and 10.0 µL of SYBR Green Ex TaqII. The cycling parameters were set as follows: Step 1: 95 ℃ for 30 s; Step 2: 40 cycles of PCR reaction (95 ℃ for 15 s, 60 ℃ for 30 s); Step 3: melt curve analysis. Fluorescent signals were collected at 65 ℃ using a CFX Connect Real-Time PCR Detection System. The relative expression levels of miR-199a-3p and FN1 were calculated according to the 2^−△△CT^ method.

### Statistical analysis

All data analyses were performed using SPSS 25.0 software. Categorical data were presented as n (%), and the chi-square test was applied for comparison. Continuous data (e.g., serum miR-199a-3p levels) were expressed as mean ± standard deviation (x̄±s), and one-way analysis of variance (ANOVA) was used for multiple group comparisons. For correlation analysis, Pearson’s correlation analysis was used when the variables (expression levels of miR-199a-3p, FN1, and DR-related indicators) conformed to a normal distribution and showed a linear relationship; Spearman’s correlation analysis was used when the variables did not conform to a normal distribution or showed a non-linear relationship. Multivariate logistic regression analysis was applied to evaluate the diagnostic value of miR-199a-3p and FN1 for DR, including their sensitivity and specificity. Two-tailed tests were used for all statistical analyses, and a P value < 0.05 was considered statistically significant. Tukey’s HSD test or Student’s t-test was used to compare the differences in miRNA expression levels between groups, with *P* < 0.05 indicating a statistically significant difference.

## Results

### Differential expression analysis of GSE102485 dataset and prediction of miR-199a-3p target genes

**Fig. 1:**
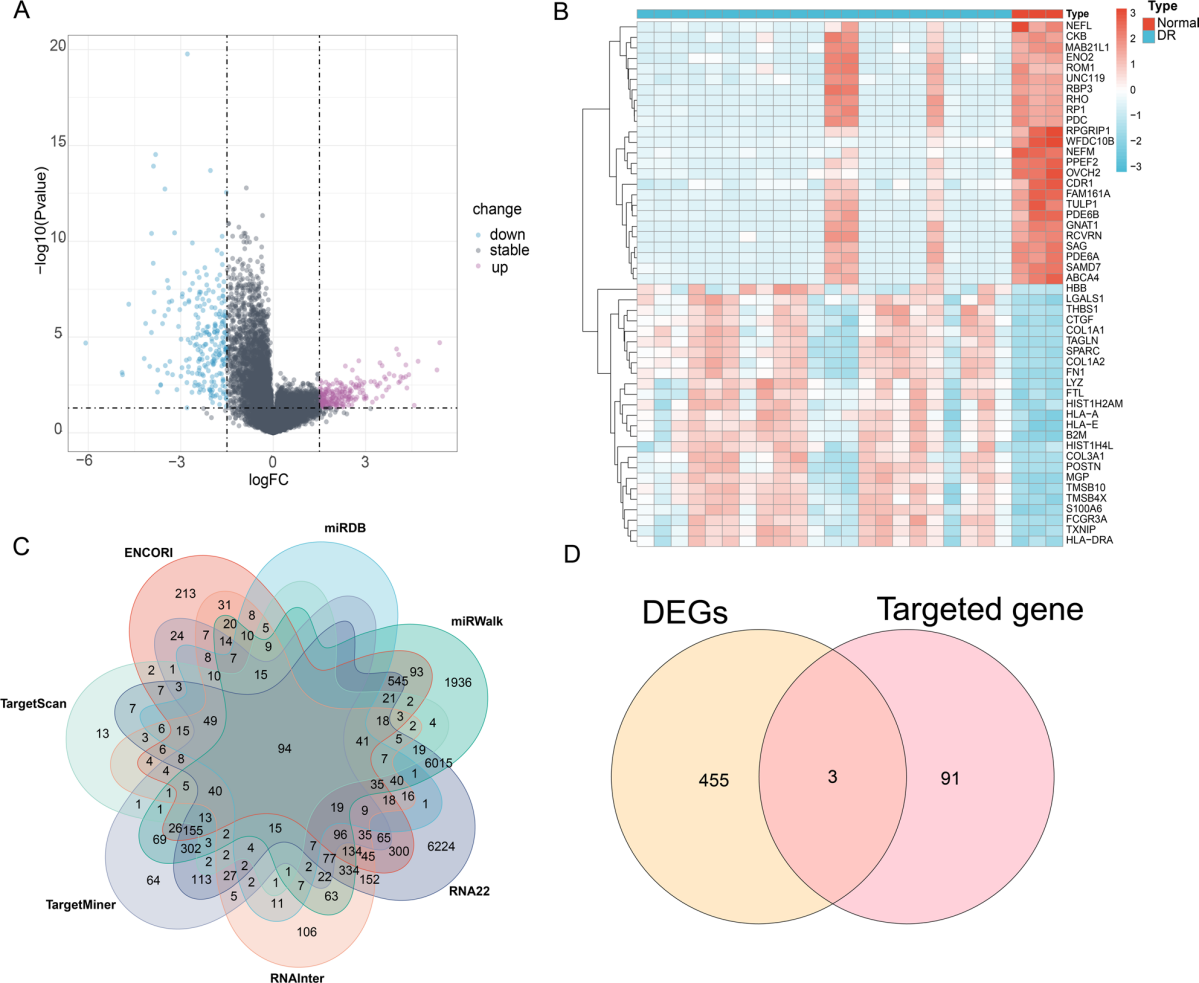
Screening of differentially expressed genes and prediction analysis of target genes. (**A**) Volcano plot of differentially expressed genes; (**B**) Heatmap of differentially expressed genes; (**C**) Venn diagram of target gene prediction; (**D**)Venn diagram of the intersection between differentially expressed genes and predicted target genes

The GSE102485 dataset comprised 3 normal samples and 22 DR samples. Differential expression analysis was performed with the screening criteria of *P* < 0.05 and |log_2_FC|>1.5, which identified a total of 458 differentially expressed genes (DEGs), including 244 significantly downregulated and 214 upregulated genes (Fig. 1A). In addition, genes were ranked by log_2_FC, and the top 50 genes with the largest fold changes were selected for heatmap construction (Fig. 1B). To screen reliable predicted target genes of miR-199a-3p, seven mainstream databases (ENCORI, miRDB, miRWalk, TargetScan, TargetMiner, RNA22 and RNAInter) were integrated, and a total of 94 target genes were commonly predicted (Fig. 1C). Furthermore, Venn diagram analysis revealed 3 overlapping genes (PHYHIPL, FN1, CALD1) between the DEGs of GSE102485 and the downstream target genes of miR-199a-3p. Subsequent analysis via the miRDB database showed that the predicted binding scores of PHYHIPL, FN1 and CALD1 among the target genes of miR-199a-3p were 75, 81 and 61, respectively. Based on these scores, FN1 was selected as the target gene for subsequent research (Fig. 1D).

### FN1 expression levels and ROC curves in the training and validation datasets

**Fig. 2:**
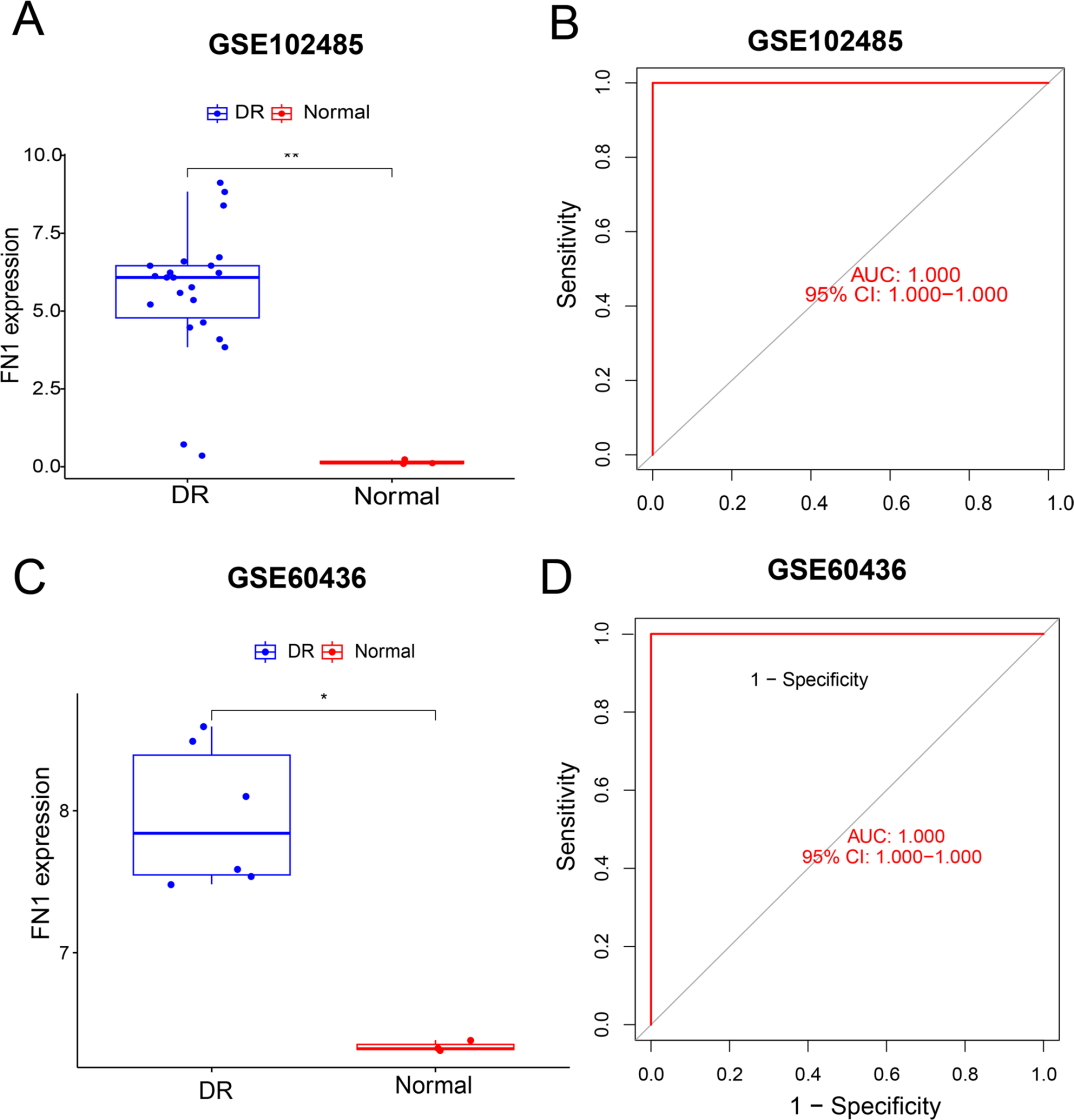
Verification of FN1 expression difference and diagnostic efficacy in DR. (**A**) Box plot of FN1 expression in the GSE102485 dataset; (**B**) ROC curve for FN1 diagnostic efficacy in the GSE102485 dataset; (**C**) Box plot of FN1 expression in the GSE60436 dataset; (**D**) ROC curve for FN1 diagnostic efficacy in the GSE60436 dataset

Moreover, the expression profile of FN1 in the DR and normal groups was plotted, and receiver operating characteristic (ROC) curves were constructed to evaluate its diagnostic efficacy. The results showed that FN1 was significantly upregulated in the DR group (Fig. 2A). The area under the curve (AUC) of FN1 for diagnosing DR reached 1.000, with a 95% confidence interval (95%CI) of 1.000 ~ 1.000, suggesting an excellent diagnostic value for DR (Fig. 2B). The GSE60436 dataset was used as an independent validation set, and repeated analysis confirmed that FN1 was still significantly upregulated in the DR group of the validation set (Fig. 2C), with an AUC of 1.000 (95%CI: 1.000 ~ 1.000) for DR diagnosis (Fig. 2D). These findings further verified the high diagnostic efficacy of FN1 for DR.

### FN-related functional and pathway enrichment analysis

**Fig. 3:**
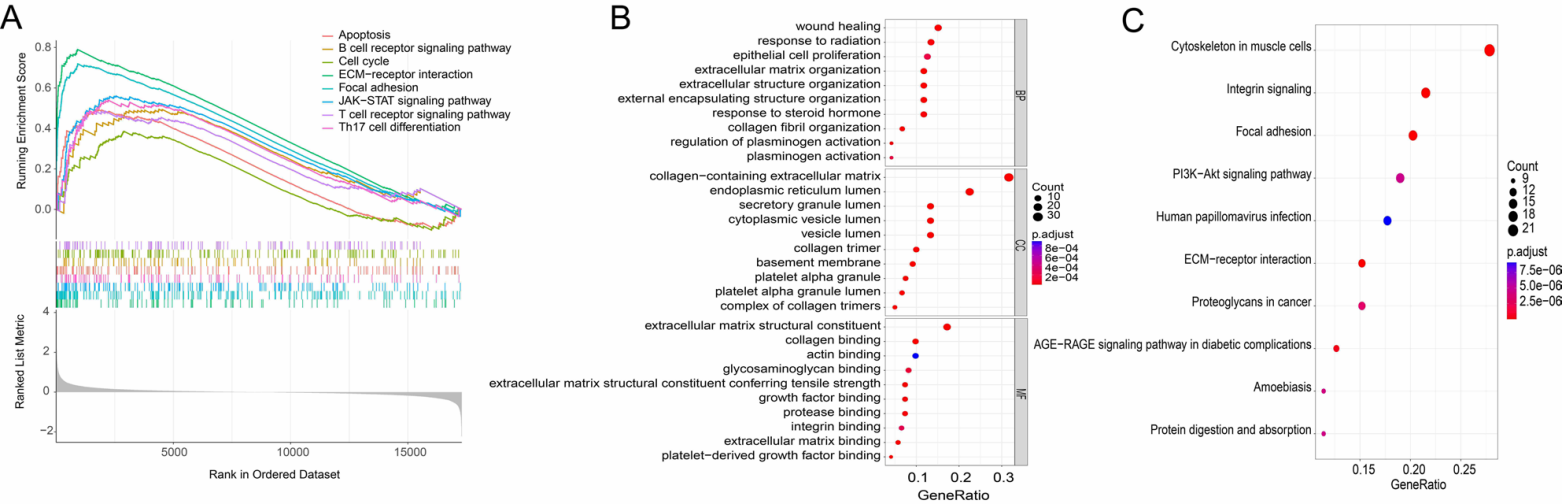
Functional enrichment analysis and pathway association network of FN1. (**A**) GSEA plot; (**B**) GO enrichment analysis plot (biological process [BP], cellular component [CC], molecular function [MF]); (**C**) KEGG enrichment plot

To explore the potential regulatory mechanism of FN1 in the development and progression of DR, GSEA was performed for FN1. The results demonstrated that in FN1 high-expression samples, multiple pathways were significantly enriched and activated, including apoptosis, B cell receptor signaling pathway, cell cycle, extracellular matrix (ECM)-receptor interaction, focal adhesion, JAK-STAT signaling pathway, T cell receptor signaling pathway and Th17 cell differentiation (Fig. 3A). Gene Ontology (GO) functional enrichment analysis showed that at the biological process (GO-BP) level, the enriched terms mainly included wound healing, epithelial cell proliferation, extracellular matrix organization and extracellular structure organization; at the cellular component (GO-CC) level, significant enrichment was observed in collagen-containing extracellular matrix, endoplasmic reticulum lumen and secretory granule lumen; at the molecular function (GO-MF) level, the enriched functions focused on extracellular matrix structural constituent, collagen binding and actin binding (Fig. 3B). Kyoto Encyclopedia of Genes and Genomes (KEGG) pathway enrichment analysis indicated that FN1-related genes were mainly involved in several pathological pathways, including integrin signaling pathway, ECM-receptor interaction, focal adhesion, advanced glycation end-product (AGE)-RAGE signaling pathway in diabetic complications and PI3K-Akt signaling pathway (Fig. 3C).

### Immune infiltration analysis and lollipop plot results

**Fig. 4:**
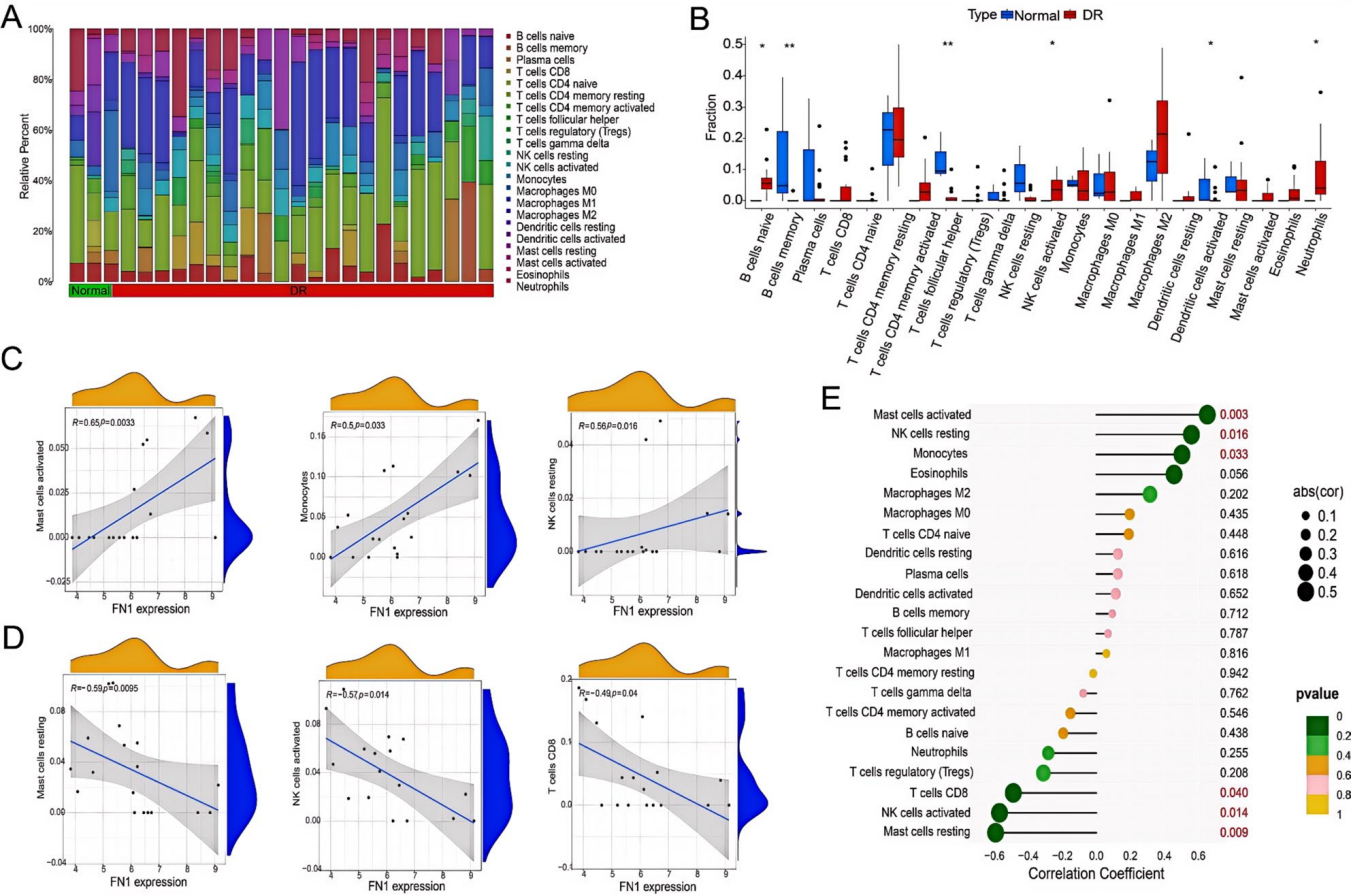
Correlation analysis of the immune microenvironment. (**A**) Heatmap of immune cell infiltration abundance; (**B**) Box plot of immune cell infiltration abundance differences; (**C-D**) Correlation scatter plots of FN1 expression and immune cell infiltration; (**E**) Correlation heatmap of FN1 expression and immune cell infiltration

Given that the aforementioned GSEA results suggested a significant association between FN1 and immune-related pathways, and immune regulation plays a core role in the pathogenesis of DR, the CIBERSORT algorithm was further applied to analyze the characteristics of immune cell infiltration in the DR and normal groups. The results showed that compared with the normal group, the DR group had significantly higher infiltration proportions of naive B cells, activated natural killer (NK) cells and neutrophils, while the infiltration proportions of memory B cells, follicular helper T cells and activated dendritic cells were significantly lower (Fig. 4A and B). Correlation analysis revealed that the expression level of FN1 was significantly positively correlated with the infiltration proportions of activated mast cells, monocytes and resting NK cells, and negatively correlated with those of resting mast cells, activated NK cells and CD8 + T cells. Lollipop plot visualization further verified these results (Fig. 4C and E).

### Results of protein-protein interaction (PPI) network analysis

**Fig. 5:**
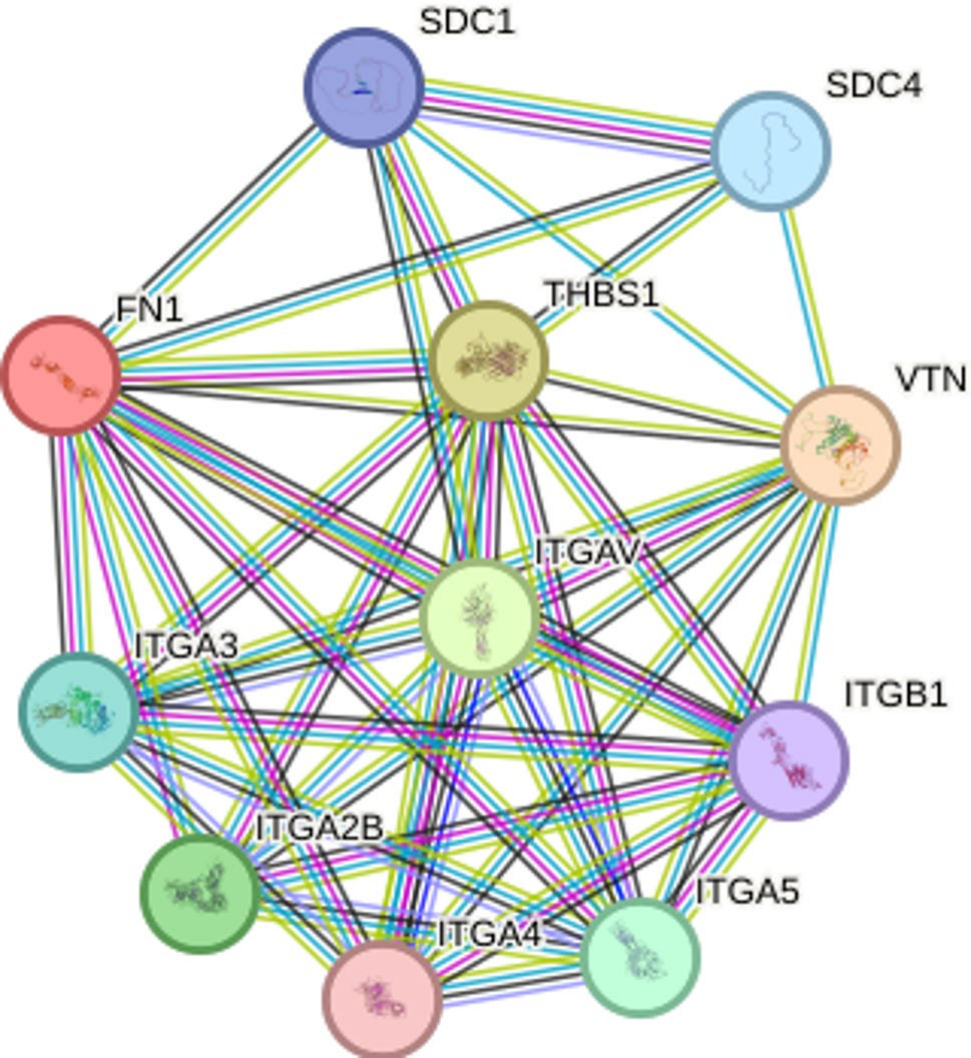
Protein-protein interaction (PPI) network mediated by the FN1 core gene

In this study, FN1 was used as the seed protein to retrieve its direct and indirect interacting proteins based on a high-confidence interaction score (with the minimum confidence score threshold set at > 0.700). This analysis aimed to reveal the complex intracellular interaction network of FN1, identify its key biological pathway partners, and provide network-level clues for an in-depth understanding of the functional mechanisms of FN1 under physiological and pathological conditions. The results showed that FN1 had targeted binding relationships with 10 proteins, including SDC1, SDC4, THBS1, VTN, ITGB1, ITGAV, ITGA3, ITGA4, ITGA5 and ITGA2B (Fig. 5).

### Comparison of clinical baseline data

There were no statistically significant differences in baseline data including age, gender ratio, intraocular pressure (IOP), triglycerides (TG), low-density lipoprotein cholesterol (LDL-C) and homeostasis model assessment of insulin resistance (HOMA-IR) among all groups (*P* > 0.05), indicating good comparability. However, significant differences were observed in body mass index (BMI), fasting blood glucose (FBG), glycosylated hemoglobin (HbA1c), total cholesterol (TC), high-density lipoprotein cholesterol (HDL-C), serum creatinine (Ser) and uric acid (UA) among the groups (*P* < 0.05). The levels of BMI, FBG, HbA1c, TC and UA in diabetes-related groups (NDR/NPDR/PDR) were significantly higher than those in the healthy control (HC) group (*P* < 0.001), and the levels of HbA1c and Ser in the PDR group were significantly higher than those in the NPDR group (*P* < 0.05). Meanwhile, the HDL-C level in diabetes-related groups was significantly lower than that in the HC group (*P* = 0.003). In addition, compared with the NDR group, the PDR and NPDR groups had significantly increased duration of type 2 diabetes, HbA1c and HOMA-IR (*P* < 0.001); the duration of diabetes and HbA1c in the PDR group were also significantly higher than those in the NPDR group (*P* < 0.05). The baseline characteristics of the enrolled subjects are shown in Table 1.

**Table 1:** Comparison of baseline clinical characteristics of study subjects in each group

Variable	HC	NDR	NPDR	PDR	*P*
Participant (n)	20	40	29	41	NA
Age (y)	59.85 ± 11.17	53.23 ± 14.11	59.00 ± 11.12	57.27 ± 13.29	0.166
BMI (kg/m²)	21.96 ± 1.66	24.66 ± 3.85^a^	21.22 ± 2.51^b^	22.24 ± 3.85^b^	0.000
IOP (mm Hg)	15.55 ± 2.28	16.45 ± 2.84	17.03 ± 3.06	16.10 ± 3.72	0.394
FBG (mmol/L)	4.80 ± 1.09	10.67 ± 3.06^a^	9.94 ± 3.24^a^	10.02 ± 3.37^a^	0.000
HbA1c (%)	5.04 ± 0.52	9.15 ± 2.16^a^	10.11 ± 2.89^a^	9.90 ± 2.65^a^	0.000
HOMA-IR	3.08 ± 0.85	22.33 ± 13.14	21.78 ± 17.97	33.74 ± 76.85	0.103
TG (mmol/L)	1.38 ± 0.39	2.50 ± 2.84	2.10 ± 1.31	1.74 ± 1.33	0.120
TC (mmol/L)	4.16 ± 0.86	5.21 ± 1.15^a^	5.31 ± 1.52^a^	4.92 ± 1.38	0.012
HDL-C (mmol/L)	1.34 ± 0.18	1.07 ± 0.30^a^	1.10 ± 0.21^a^	1.09 ± 0.31^a^	0.003
LDL-C (mmol/L)	3.06 ± 0.33	2.98 ± 0.87	3.09 ± 1.05	2.81 ± 1.18	0.628
Ser (µmol/L)	58.80 ± 7.25	65.09 ± 22.71	71.74 ± 31.79	175.55 ± 266.53^abc^	0.003
UA (µmol/L)	218.25 ± 83.46	290.48 ± 71.54^a^	329.38 ± 92.53^a^	391.44 ± 124.64^abc^	0.000
Gender (M/F)	7/13	24/16	16/13	27/14	0.142

Note.:Values are presented as mean ± SD or number (%). Statistical analyses were performed using one-way ANOVA for continuous variables and Chi-square test for categorical variables.^a^*P*<0.05, ^b^*P*<0.05, ^c^*P*<0.05 versus HC, NDR, NPDR groups, respectively

BMI: Body mass index; FBG: Fasting plasma glucose; HbA1c: Glycated hemoglobin; HC: Healthy control; HDL-C: High-density lipoprotein cholesterol; HOMA-IR: Homeostatic model assessment of insulin resistance; IOP: Intraocular pressure; LDL-C: Low-density lipoprotein cholesterol; NA: No available; NDR: No diabetic retinopathy; NPDR: Non-proliferative diabetic retinopathy; PDR: Proliferative diabetic retinopathy; Ser: Serum creatinine; TC: Total cholesterol; TG: Triglyceride; UA: Uric acid

### Comparison of miR-199a-3p and FN1 expression levels in serum and aqueous humor among different groups

**Fig. 6:**
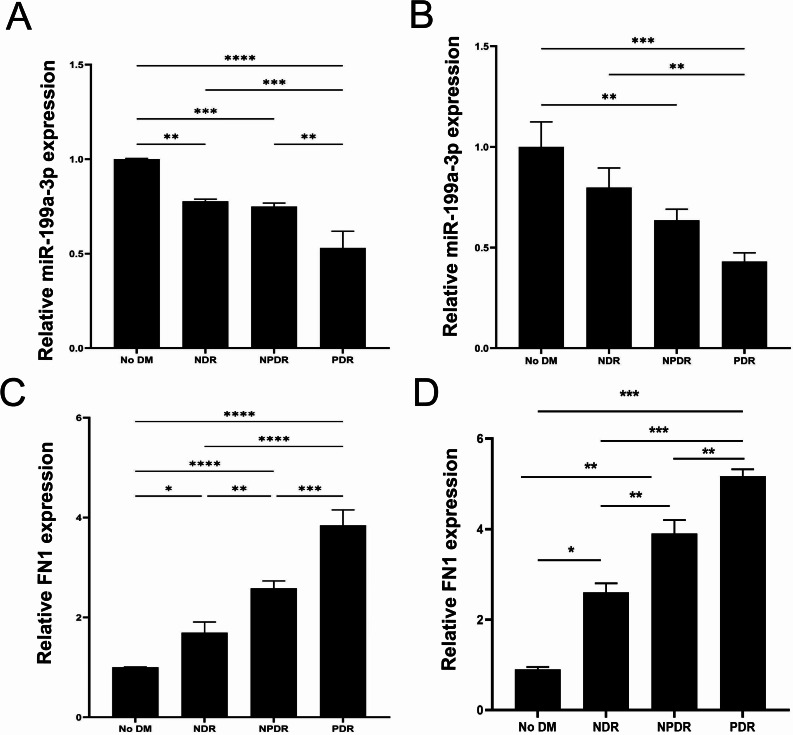
Expression changes of miR-199a-3p and FN1 during DR progression. Expression of miR‑199a‑3p in serum (**A**) and aqueous humor (**B**), and expression of FN1 in serum (**C**) and aqueous humor (**D**). Groups included: No DM, healthy control subjects; NDR, diabetic patients without retinopathy; NPDR, non‑proliferative diabetic retinopathy; PDR, proliferative diabetic retinopathy. Sample sizes were n = 20 for the No DM and NDR groups, and n = 10 for the NPDR and PDR groups. Comparisons among multiple groups were performed using one‑way ANOVA, followed by LSD post‑hoc test for pairwise comparisons. *P < 0.05, **P < 0.01, ***P < 0.001, ****P < 0.0001 vs. the indicated group

The relative expression levels of miR-199a-3p showed significant differences among all study groups (Fig. 6A and B). In serum samples (Fig. 6A), compared with the HC group, the expression levels of miR-199a-3p in the non-diabetic retinopathy (NDR) group and non-proliferative diabetic retinopathy (NPDR) group were significantly decreased (both *P* < 0.01), with a more significant reduction in the proliferative diabetic retinopathy (PDR) group (*P* < 0.001). The same trend was observed in aqueous humor samples (Fig. 6B): the expression level of miR-199a-3p showed a gradual downregulation with the progression of DR (NDR→NPDR→PDR). Intergroup comparisons showed statistically significant differences between the NDR and NPDR groups (*P* < 0.001), NDR and PDR groups (*P* < 0.01), as well as NPDR and PDR groups (*P* < 0.01).

The relative expression levels of FN1 showed a significant increasing trend among the four groups (Fig. 6C and D). In serum samples (Fig. 6C), compared with the HC group, the expression level of FN1 in the NDR group was significantly increased (*P* < 0.05), with a more significant elevation in the NPDR group (*P* < 0.001) and an extremely significant increase in the PDR group (*P* < 0.0001). In aqueous humor samples (Fig. 6D), intergroup comparisons revealed statistically significant differences in FN1 expression levels between the NDR and NPDR groups (*P* < 0.05), NDR and PDR groups (*P* < 0.001), and NPDR and PDR groups (*P* < 0.001). These results suggested that the expression of FN1 increased gradually with the progression of DR, both in serum and aqueous humor.

## Discussion

Diabetic retinopathy (DR) is the most common and severe microvascular complication in patients with diabetes mellitus, and it has emerged as one of the leading causes of visual impairment in the working-age population worldwide. Its pathological process involves retinal microvascular damage and neuronal injury, with no obvious clinical symptoms in the early stage. However, as the disease progresses, complications such as diabetic macular edema and proliferative diabetic retinopathy may develop, leading to significant visual loss and even blindness. With the rising global prevalence of diabetes, the prevention, early diagnosis and effective intervention of DR have become an urgent clinical and public health priority. Current diagnostic approaches rely heavily on imaging examinations, yet the lack of sensitive and specific molecular biomarkers limits the achievement of early disease identification and individualized treatment [13, 14]. In addition, the pathogenesis of DR is complex, involving multiple molecular pathways such as inflammatory response, fibrosis and extracellular matrix (ECM) remodeling [15, 16]. Therefore, in-depth exploration of its key regulatory factors is of great significance for elucidating the pathological essence of the disease and developing novel therapeutic strategies.

In this study, we systematically screened DR-related differentially expressed genes (DEGs) from the public Gene Expression Omnibus (GEO) database, with a specific focus on the expression characteristics and correlation of miR-199a-3p and its potential target gene FN1. Through an integrated approach of comprehensive bioinformatics analysis and clinical sample validation, we revealed the dynamic expression patterns of miR-199a-3p and FN1 in DR and their significant association with disease severity, suggesting that both may play critical regulatory roles in the pathological progression of DR. Furthermore, miR-199a-3p was negatively correlated with glycemic control indicators, while FN1 showed a positive correlation, implying their potential mechanisms in diabetic metabolic disorders and retinal injury. The findings of this study not only provide a novel perspective for understanding the molecular pathogenesis of DR but also lay a foundation for the future development of biomarkers and the design of targeted intervention strategies. Subsequent studies will further investigate the functions of these key molecules and the mechanisms of their associated signaling pathways.

Through differential expression analysis of the GSE102485 and GSE60436 datasets, we identified a total of 458 DEGs, including 214 upregulated and 244 downregulated genes, and visualized the 50 genes with the most significant expression changes via heatmaps. This finding not only enriches the repertoire of DR-related genes but also provides candidate targets for subsequent molecular mechanism research. Accumulating evidence has demonstrated that diabetes-induced retinal injury involves a complex gene expression regulatory network, which exerts key effects particularly in ECM remodeling, inflammatory response and angiogenesis. The combined analysis of multiple datasets in this study effectively reduced the batch effects that may exist in a single dataset, thus improving the reliability and generalizability of the screening results [17]. In addition, the enrichment of the identified DEGs in processes such as signal transduction, cell adhesion and immune regulation suggests their potential involvement in the multi-level regulatory mechanisms of DR, expanding the current understanding of the molecular pathology of DR. In summary, the systematic identification of DEGs provides a solid foundation for dissecting the molecular network of DR.

Venn diagram analysis revealed the overlap between DEGs in GSE102485 and the predicted target genes of miR-199a-3p, among which FN1 exhibited the strongest correlation, indicating that FN1 may play a central role in DR. As a key component of the ECM, FN1 regulates cell adhesion, migration and fibrogenesis, and its abnormal expression has been confirmed in various diabetes-related complications. By targeting and regulating FN1 expression, miR-199a-3p may affect retinal ECM remodeling and fibrosis, thereby participating in the pathological progression of DR [18]. Notably, the specific regulatory mechanism of the miR-199a-3p-FN1 axis in DR remains to be further explored, especially its potential crosstalk with classical signaling pathways closely related to DR pathogenesis, such as TGF-β and NF-κB pathways. Existing studies have confirmed that TGF-β pathway is a key regulator of retinal fibrosis and vascular homeostasis in DR [19], and it can promote the expression of ECM-related genes to accelerate retinal structural damage; while NF-κB pathway, as a core inflammatory regulatory pathway, mediates hyperglycemia-induced retinal inflammatory response and immune cell infiltration. We hypothesize that miR-199a-3p may regulate FN1 expression by targeting the TGF-βor NF-κB pathway: on the one hand, miR-199a-3p may inhibit the activation of TGF-β pathway, thereby reducing the transcriptional activation of FN1 and alleviating retinal ECM deposition; on the other hand, miR-199a-3p may suppress the NF-κB pathway-mediated inflammatory response, indirectly regulating FN1 expression and further improving the retinal inflammatory microenvironment. However, the specific binding sites and regulatory mode between miR-199a-3p and these pathways, as well as whether there is a synergistic or antagonistic effect between the two pathways in the regulation of FN1, need to be verified by subsequent in vitro and in vivo experiments.Different from previous studies focusing on the regulatory role of miRNAs in inflammation and angiogenesis during diabetic retinal injury, this study is the first to systematically reveal the potential regulatory relationship of the miR-199a-3p-FN1 axis using bioinformatics methods, providing a new insight into the pathogenesis of DR. Moreover, the function of FN1 in fibrosis and ECM remodeling is recognized as a critical factor in retinal structural changes in DR, supporting its rationality as a therapeutic target [20]. Therefore, the interaction mechanism between miR-199a-3p and FN1 holds important research and clinical value.

Signaling pathway enrichment analysis revealed that the DEGs were mainly involved in multiple pathways closely associated with diabetes and DR, such as oxidative stress response, inflammatory signaling pathways and ECM remodeling. Notably, the significant enrichment of the TGF-β and NF-κB signaling pathways echoes their dual roles in DR pathology: TGF-β not only regulates retinal vascular homeostasis but also participates in retinal fibrosis and inflammation [20], while NF-κB, as a core regulatory factor of inflammation, mediates hyperglycemia-induced immune responses in the retinal microenvironment [21]. The results of this study are highly consistent with existing literature reports on the effects of these signaling pathways on DR progression. Furthermore, by integrating miRNA target gene prediction and differential expression data, we further clarified the involvement of key genes in these pathways, providing systematic molecular evidence for unraveling the complex regulatory network of DR. In addition, pathway analysis suggested that the crosstalk between oxidative stress and apoptosis plays an important role in DR, supporting therapeutic strategies targeting these signaling pathways [22]. Thus, systematic signaling pathway analysis deepens the understanding of the molecular mechanisms underlying DR.

Serum miR-199a-3p levels were negatively correlated with fasting blood glucose, glycosylated hemoglobin and disease duration, whereas FN1 showed a positive correlation, revealing that both may influence DR progression by regulating inflammation and metabolic disorders. Inflammatory response is considered a crucial driver of DR pathogenesis, and miR-199a-3p may alleviate inflammatory responses by inhibiting the NF-κB signaling pathway, thereby exerting a protective effect on the retina [21]. In contrast, the upregulation of FN1 promotes ECM deposition and fibrosis, exacerbating the inflammatory microenvironment of retinal injury [20]. In addition, although immune infiltration analysis was conducted in this study, the relationship between FN1 expression and the functional status of immune cells (especially M1/M2 microglia) was not deeply discussed, which is a deficiency to be supplemented. Microglia, as the main immune cells in the retina, exist in two functional phenotypes: M1 type (pro-inflammatory phenotype) and M2 type (anti-inflammatory phenotype). The imbalance of M1/M2 microglia polarization is closely related to the progression of DR, and M1 microglia-mediated pro-inflammatory response can further aggravate retinal vascular injury and neuronal damage, while M2 microglia can secrete anti-inflammatory factors to promote tissue repair [23]. We speculate that FN1 may be involved in regulating the polarization of M2/M1 microglia: the high expression of FN1 in DR may induce the polarization of microglia to M1 type, enhance the release of pro-inflammatory cytokines (such as TNF-α, IL-6), and further amplify the retinal inflammatory response; on the contrary, the downregulation of FN1 may promote the polarization of microglia to M2 type, thereby alleviating retinal inflammation and tissue damage. In addition, FN1, as an ECM component, may also bind to surface receptors of immune cells (such as integrins), regulate the adhesion and migration of immune cells, and further affect their functional status [24]. The specific regulatory relationship between FN1 and M2/M1 microglia polarization, as well as the underlying molecular mechanism, need to be further verified by subsequent cell experiments.This expression pattern was validated in clinical samples in the present study, which echoes the critical role of immune regulation in DR development and is consistent with previous research on inflammation-mediated pathological mechanisms of DR [15]. Additionally, the expression changes of miR-199a-3p and FN1 may also affect retinal vascular permeability and cell apoptosis, thereby promoting disease progression [25]. In conclusion, the opposing regulatory effects of miR-199a-3p and FN1 in the immunopathology of DR identify novel molecular therapeutic targets.

In this study, qPCR technology was used to validate the correlation between the expression of miR-199a-3p and FN1 and DR severity in clinical serum and aqueous humor samples, further consolidating the accuracy of the bioinformatics results. This approach not only enhances the clinical translational potential of the data but also demonstrates the feasibility of these two molecules as biomarkers for the early diagnosis of DR [20]. Compared with traditional imaging-based diagnostic methods, the application of molecular biomarkers is expected to enable earlier and more accurate disease monitoring [13]. Furthermore, qPCR validation results showed an inverse expression pattern between miR-199a-3p and FN1, supporting the functional relevance of their regulatory relationship, which is consistent with previous studies on miRNA regulation in diabetes complications [18]. Although the current sample size is limited, this validation lays a foundation for the future development of diagnostic tools and therapeutic strategies based on miRNAs and their target genes, which is expected to advance molecular diagnosis and personalized treatment of DR.

This study has several limitations, mainly related to the sample size and the diversity of data sources. First, although multiple datasets were analyzed from the GEO database, the number of included samples was relatively small, which may reduce the reliability of statistical results and limit a comprehensive understanding of the relationship between miR-199a-3p and FN1. In addition, the heterogeneity of the datasets may introduce batch effects, affecting the screening results of DEGs and thus exerting an adverse impact on the external generalizability of the study findings. These limitations may to some extent obscure the real roles of miR-199a-3p and FN1 in DR.

In conclusion, this study successfully identified 458 DEGs and, more importantly, revealed the correlation between miR-199a-3p and FN1, providing a novel perspective for mechanistic research on diabetic retinopathy. This finding not only offers theoretical support for the future development of biomarkers but also points out the direction for early diagnosis and targeted therapy of DR. In response to the research deficiencies pointed out in the review comments, future studies will focus on two key aspects: first, we will further explore the specific regulatory mechanism of the miR-199a-3p-FN1 axis, especially its crosstalk with TGF-βand NF-κB signaling pathways, through in vitro cell experiments and in vivo animal models, to clarify the molecular basis of their involvement in DR progression; second, we will deeply investigate the relationship between FN1 expression and the functional status of immune cells (such as M2/M1 microglia), clarify whether FN1 can regulate DR progression by affecting microglia polarization, and supplement the immune-related regulatory mechanism of the miR-199a-3p-FN1 axis. Future studies should consider expanding the sample size and combining wet experiment validation to further confirm the clinical application potential of these biomarkers and promote the development of early intervention strategies for DR.

## Conclusion

This study confirmed that miR-199a-3p and FN1 in serum and aqueous humor are closely correlated with the severity of type 2 DR in the Chinese population. MiR-199a-3p was progressively downregulated with DR progression and negatively correlated with glycemic indicators, while FN1 was significantly upregulated and positively correlated with such indicators. Bioinformatics analysis identified FN1 as the key target gene of miR-199a-3p, with the miR-199a-3p-FN1 axis involved in DR-related pathological processes like ECM remodeling and immune infiltration. Both molecules exhibited good diagnostic efficacy for DR. This study clarifies their regulatory role in DR pathogenesis, suggesting their potential as non-invasive diagnostic/prognostic biomarkers and providing a novel molecular target for DR targeted therapy. Limitations include small sample size, and further multi-center and functional experiments are needed for validation.

## Data Availability

The data that support the findings of this study are available from the authors.
